# Inflammation in Chemotherapy-Induced Cardiotoxicity

**DOI:** 10.1007/s11886-024-02131-5

**Published:** 2024-10-08

**Authors:** Elizabeth Hutchins, Eric H. Yang, Ashley F. Stein-Merlob

**Affiliations:** 1 Division of Cardiology, Department of Medicine, University of California Los Angeles, Los Angeles, CA USA; 2 Cardio-Oncology Program, Division of Cardiology, Department of Medicine, University of California Los Angeles, Los Angeles, CA USA

**Keywords:** Cardio-oncology, Anthracyclines, Cancer treatment related cardiac dysfunction, Inflammation, Doxorubicin, Cardioprotection

## Abstract

**Purpose of Review:**

In this review we describe the role of inflammation in chemotherapy-induced cardiotoxicity with a particular focus on anthracycline-induced cardiomyopathy (AIC). First, we discuss inflammation associated with anthracyclines at a cellular level. Next, we discuss the clinical implications of these inflammatory mechanisms for early detection and cardioprotective strategies in patients undergoing anthracycline treatment.

**Recent Findings:**

Key inflammatory pathways identified in AIC include cytokine release, upregulation of the innate immune system via toll-like receptors, and activation of the inflammasome. Emerging evidence suggests a role for inflammatory biomarkers in detecting subclinical AIC. Advanced imaging techniques, such as cardiac PET with novel tracers targeting inflammation, may enhance early detection. Both traditional cardioprotective strategies and novel anti-inflammatory therapies show potential in preventing and treating AIC.

**Summary:**

Understanding the inflammatory mechanisms involved in AIC provides new opportunities for early detection and targeted cardioprotective strategies in patients undergoing anthracycline treatment and informs our understanding of other forms of chemotherapy-induced cardiotoxicity.

## Introduction

Advances in oncologic care have rapidly reduced mortality in cancer patients. With improved survival there is increased focus on cardiovascular side effects of cancer treatments, collectively termed cancer therapy related cardiac dysfunction (CTRCD). Understanding the underlying mechanisms of CTRCD is critical for developing improved cardioprotective protocols and novel, safer treatments in the future. Additionally, as the mechanisms of cardiotoxicity are better understood, they provide important insights into myocardial physiology and particularly myocardial responses to stressors.

**Fig. 1:**
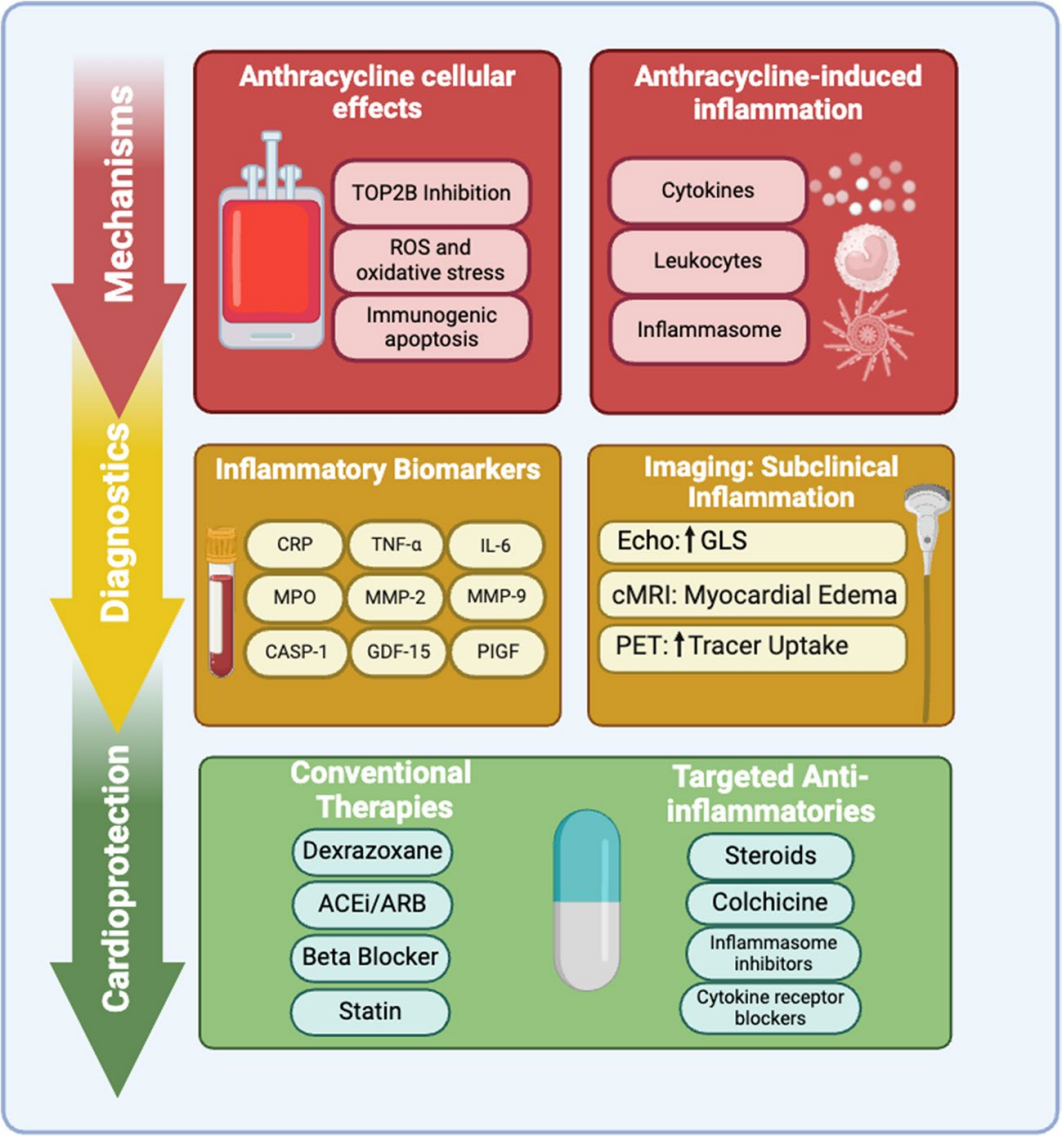
Anthracycline-induced Inflammation: Mechanisms, Diagnostics, Cardioprotection.  Abbreviations: TOP2B: Topoisomerase II beta; ROS: Reactive Oxygen Species; CRP: C-reactive Protein; TNF-α: Tumor Necrosis Factor-alpha; IL-6: Interleukin-6; MMP-2: Matrix Metalloproteinase-2; MMP-9: Matrix Metalloproteinase-9; MPO: Myeloperoxidase; CASP-1: Caspase-1; GDF-15: Growth Differentiation Factor 15; PIGF: placental growth factor; GLS: Global Longitudinal Strain; cMRI: Cardiac Magnetic Resonance Imaging; PET: Positron Emission Tomography; ACEi: Angiotensin-Converting Enzyme Inhibitor; ARB: Angiotensin Receptor Blocker; NLRP3: Nucleotide-binding Oligomerization Domain-like Receptor Family Pyrin Domain Containing 3

Inflammation is a complex set of biological responses to harmful stimuli, acting as an adaptive mechanism that mobilizes the immune system to counter these threats. However, prolonged or chronic inflammation can become maladaptive, increasing the risk of disease. Inflammation underlies many forms of CTRCD and, in particular, plays a crucial role in the development of cardiomyopathy from anthracyclines, known as anthracycline-induced cardiomyopathy (AIC). In this article, we describe the role of inflammation in AIC, which is the most well-characterized cause of CTRCD. This is summarized in Fig. 1**.** First, we examine the biologic mechanisms of anthracycline-induced inflammation at a cellular level. Next, we discuss the clinical evidence for AIC-induced inflammation, focusing on laboratory biomarkers and cardiac imaging. Finally, we discuss the role of medications in targeting inflammation associated with AIC. Understanding the role of inflammation in AIC provides insight into all forms of CTRCD and has the potential to improve the long-term cardiovascular health of patients with cancer.

## Biological Mechanisms of Inflammation in Anthracycline-Induced Cardiomyopathy (AIC)

**Fig. 2:**
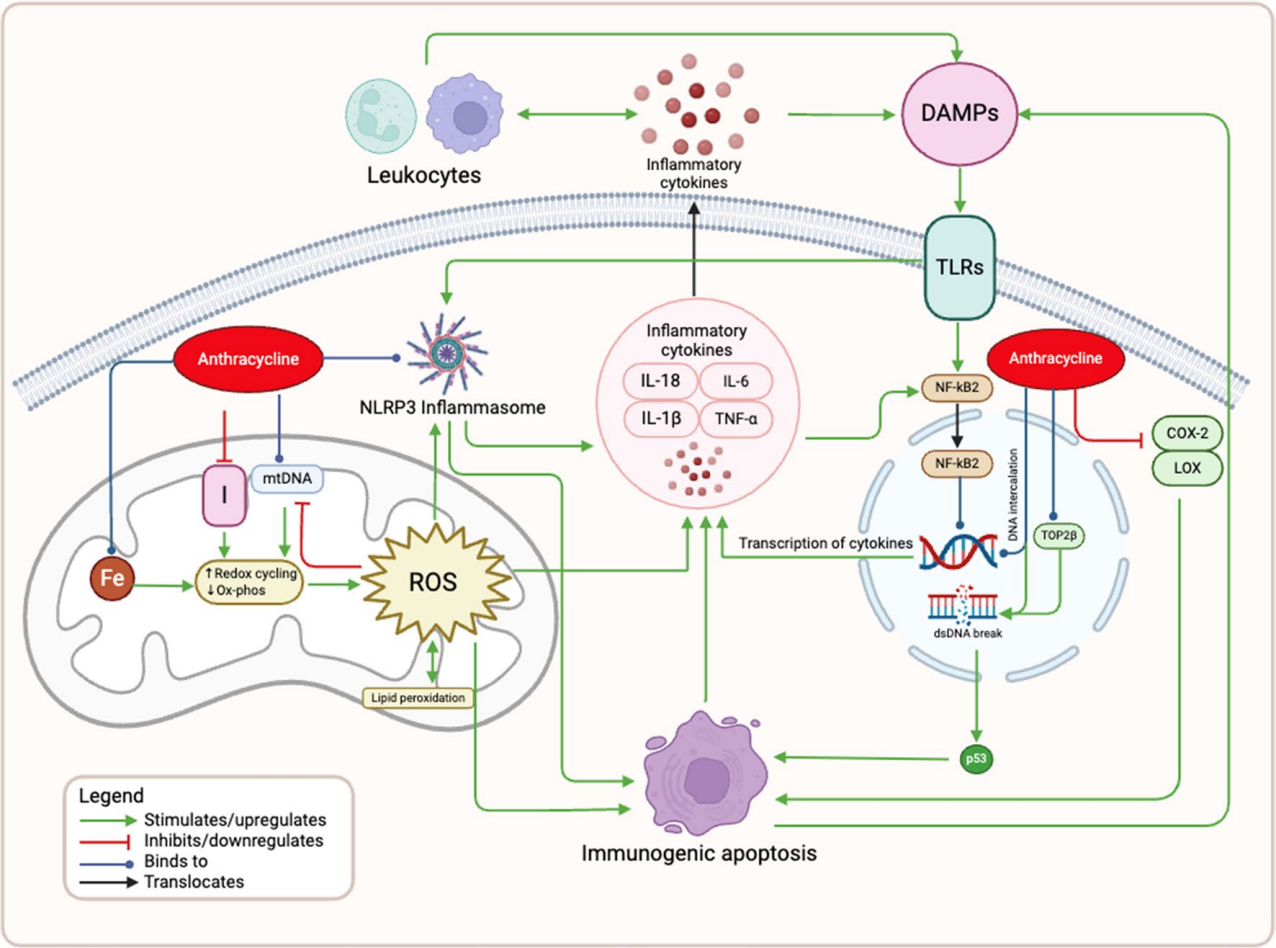
Mechanisms of Anthracycline-Induced Inflammation. Anthracyclines exert their toxic effects primarily by inhibiting topoisomerase II beta (TOP2B) and intercalating with DNA, leading to double-strand DNA (dsDNA) breaks and the activation of the tumor suppressor protein p53, which promotes immunogenic apoptosis. Anthracyclines also disrupt mitochondrial function by binding to complex I (NADH dehydrogenase) and mitochondrial DNA (mtDNA), leading to excessive reactive oxygen species (ROS) production, lipid peroxidation, and mitochondrial damage. The generation of ROS triggers the activation of the NLRP3 inflammasome and the release of inflammatory cytokines. These cytokines, in turn, recruit leukocytes to the myocardium and further exacerbate inflammation. Additionally, anthracyclines induce the release of damage-associated molecular patterns (DAMPs), which activate Toll-like receptors (TLRs) and lead to the nuclear translocation of NF-κB, driving the transcription of pro-inflammatory cytokines. The inhibition of cyclooxygenase-2 (COX-2) and lipoxygenase (LOX) by anthracyclines further diminishes anti-inflammatory defenses, amplifying the inflammatory response. Abbreviations: TOP2B: Topoisomerase II beta; ROS: Reactive Oxygen Species; mtDNA: Mitochondrial DNA; NLRP3: Nucleotide-binding Oligomerization Domain-like Receptor Family Pyrin Domain Containing 3; IL-1β: Interleukin-1 beta; IL-6: Interleukin-6; IL-18: Interleukin-18; TNF-α: Tumor Necrosis Factor-alpha; DAMPs: Damage-Associated Molecular Patterns; TLRs: Toll-like Receptors; NF-κB: Nuclear Factor kappa-light-chain-enhancer of activated B cells; dsDNA: Double-Strand DNA; p53: Tumor Suppressor Protein p53; COX-2: Cyclooxygenase-2; LOX: Lipoxygenase

Anthracyclines are potent cytotoxic antibiotics integral to many anti-cancer regimens. However, they are associated with a dose-dependent risk of cardiomyopathy in a significant subset of patients. For instance, a recent cohort study reported a 9% incidence of cardiotoxicity with a median follow-up of 5.2 years [1]. The risk is dose-dependent, with a 26% incidence of cardiotoxicity when the dose exceeds 550 mg/m^2^ [2]; currently any dose above 240 mg/m^2^ is considered high-risk for development of AIC [3]. Early studies of AIC primarily focused on oxidative stress; however, the pathophysiology of AIC involves multiple interconnected cellular mechanisms, with inflammation playing a crucial role. The detailed mechanisms are outlined below and depicted in Fig. 2. A thorough understanding of these pathways may reveal strategies for the early detection and treatment of AIC.

### Direct Cellular Cardiotoxic Effects of Anthracyclines

Doxorubicin, the most widely used anthracycline, accumulates at a cellular level primarily in the nuclei and mitochondria [4]. The primary nuclear target of doxorubicin is topoisomerase-IIβ (TOP2B) [5], an enzyme that prevents nuclear DNA breaks. When doxorubicin binds to TOP2B, it inhibits the enzyme’s ability to bind to DNA and prevent ligation. Additionally, doxorubicin intercalates with DNA strands, leading to DNA breaks [6]. The resultant DNA damage activates pro-apoptotic signaling pathways, notably those involving p53, resulting in cell apoptosis [5, 7].

In the mitochondria, doxorubicin binds to complex I (NADH dehydrogenase) of the electron transport chain [8, 9], and to mitochondrial free iron creating highly reactive iron-anthracycline complexes [10]. Additionally, doxorubicin intercalates with mitochondrial DNA (mtDNA) [11]. These interactions stimulate excessive redox cycling and disrupt mitochondrial function, leading to the uncoupling of the electron transport chain and the formation of reactive oxygen species (ROS) [12]. ROS cause oxidative stress, leading to damage to the mitochondria through processes such as lipid peroxidation of the mitochondrial membrane [10] [13]. Severe oxidative damage and ATP depletion can result in cardiomyocyte death.

### Activation of the Immune System by Anthracyclines

The actions of doxorubicin in the nucleus and mitochondria are inextricably linked with cardiac inflammation via activation of the immune system. Severe oxidative damage and ATP depletion, as described above, result in cell death characterized by cell swelling, membrane rupture, and inflammation [14]. While traditional forms of apoptosis do not induce an inflammatory response; doxorubicin induces immunogenic forms of cell death, including pyroptosis (discussed below) and necrosis, which trigger an inflammatory response from the immune system [14] [15]. ROS and oxidative stress also trigger an inflammatory response through the production of inflammatory cytokines [16]. These cytokines attract immune cells, such as macrophages and neutrophils, to the myocardium [17]. These immune cells release additional inflammatory mediators, ROS, and proteolytic enzymes, exacerbating myocardial injury.

Doxorubicin-induced cell death leads to the production of key inflammatory mediators, including damage-associated molecular patterns (DAMPs) and endotoxins. DAMPs are signals of host cellular distress [18], released by both doxorubicin-killed tumor cells [19] as well as macrophages activated by doxorubicin-induced inflammation [20] [21]. Endotoxins are toxins released by gram-negative bacteria, and have been shown to enter the circulation due to doxorubicin-induced increases in intestinal permeability [22]. DAMPs and endotoxins upregulate and activate Toll-like receptors (TLRs) [23], which are key mediators of the innate immune system’s inflammatory response. Activation of the TLR intracellular signaling leads to nuclear localization of pro-inflammatory transcription factor NF-kB [24], which drives the transcription of genes encoding pro-inflammatory cytokines, namely tumor necrosis factor-alpha (TNF-α), interleukin-1 beta (IL-1β), interleukin-6 (IL-6), and interleukin-18 (IL-18) [25]. TNF-α further amplifies this response by stimulating NF-kB in a feed-forward mechanism [26]. In animal models, both TLR2 knockout and antagonism have been shown to attenuate doxorubicin-induced inflammation and subsequent cardiac injury [20, 23].

Another key mechanism of inflammation in AIC is increased activation of the NOD-like receptor protein 3 (NLRP3) inflammasome in cardiomyocytes by doxorubicin [27]. The NLRP3 inflammasome is a large tripartite protein consisting of an intracellular sensor (NLRP3), an adaptor, and an effector (caspase 1) [28]. The inflammasome functions through two critical steps, priming and activation. Priming leads to upregulation of the expression of component proteins, which is typically triggered by DAMP and pathogen-associated molecular patterns (PAMP) binding to TLRs. Next, activation triggers oligomerization into the NLRP3 inflammasome to activate caspase 1, which in turn cleaves pro-IL-1β and pro-IL-18 leading to inflammatory cytokine release [29]. The inflammasome is activated by a wide range of stimuli, particularly products of cellular stress, including release of ROS. Doxorubicin has been shown in preclinical models to increase activation of the NLRP3 inflammasome [27]. In addition to direct release of cytokines, the NLRP3 signaling pathway leads to an inflammatory programmed cell death called pyroptosis, leading to release of inflammatory factors and cell contents [30]. Pyroptosis is an important mechanism for the anticancer properties of anthracyclines but contributes to the off-target side effects. Through this form of cell death, release of DAMPs and cytokines cause an inflammatory cycle that leads to further myocardial damage and cardiotoxicity. In addition, activation of the inflammasome inhibits cardiomyocyte autophagy, a non-inflammatory, protective form of cell death [31].

In addition to triggering the immune system as described above, anthracyclines can inhibit the natural anti-inflammatory mechanisms within the myocardium. Anthracyclines inhibit cyclooxygenase and lipoxygenase enzymes, reducing the availability of anti-inflammatory mediators like prostacyclins in the heart. This reduction of anti-inflammatory protection mechanisms exacerbates inflammation and contributes to cardiac damage associated with anthracyclines [32].

### Myocardial Effects of Inflammation

While myocyte death from the above-described mechanisms lead to a significant change in myocardial function, inflammatory cytokines also lead to myocyte dysfunction in the surviving myocytes. Inflammatory cytokines, including TNF-α, IFN-γ, IL-6, IL-8 and IL-1β cause changes in cytoskeleton structure and mitochondrial function, which can significantly affect the contractile properties without inducing cell death, which was previously thought to be the driver of cardiac dysfunction [19, 33]. This is similar to the well described mechanism of sepsis-induced cardiomyopathy and cardiotoxicity seen in cytokine release syndrome with chimeric antigen receptor (CAR) T-cell therapy [34].

## Novel Methods of Detection for Cardiotoxicity

The emerging understanding of the role of inflammation in CTRCD offers promising new approaches to the early detection and treatment of cardiotoxicity. The current mainstay of detection of cardiotoxicity is serial echocardiography with global longitudinal strain (GLS) [3]. In current practice, there is also a role for laboratory biomarkers, particularly troponin and B-type natriuretic peptide (BNP), for additional monitoring in patients at increased risk for cardiotoxicity. Notably, high sensitivity troponin T has been found to be increased in patients who develop CTRCD from anthracyclines and trastuzumab [35]. As our understanding of the inflammatory underpinnings of AIC grows, there is potential for more advanced laboratory biomarkers to detect AIC in its subclinical state. Similarly, with advancements in imaging techniques, there is new opportunity to develop earlier markers of cardiotoxicity to ensure prompt detection and treatment. However, it is important to note that when detecting inflammation in AIC, imaging and cardiac biomarkers are not specific to AIC and can represent inflammation from other sources, including tumor itself. Increased understanding of the role of inflammation in AIC at a cellular level, beyond what is described above, may open possibilities for additional clinical diagnostic methods.

### Inflammatory Biomarkers

Biomarkers are an important potential avenue for the early diagnosis of CTRCD as they may provide a cost effective and reproducible method of early detection. However, the utility of inflammatory biomarkers in detection of cardiotoxicity is complex and significantly confounded by the overall inflammatory milieu of the underlying malignancy. Preclinical and clinical studies have demonstrated that, even prior to the initiation of anthracycline, plasma levels of inflammatory markers, such as cytokines and metalloproteinases, are elevated in the presence of tumor. In a mouse model of colorectal cancer, elevated IL-6 and IL-8 correlated with myocardial dysfunction even prior to doxorubicin administration [33]. Similarly, a clinical study of patients with breast cancer demonstrated that inflammatory markers, including myeloperoxidase (MPO), IL-6, and matrix metalloproteases (MMP-2 and MMP-9), were significantly elevated compared to normal controls prior to anthracycline administration [36].

The literature presents mixed findings regarding serum biomarker changes after the initiation of anthracycline therapy. While some biomarkers increase post-anthracycline chemotherapy and potentially signal cardiotoxicity, others decrease. In the previously mentioned study, levels of TOP2β, MPO, MMP-2, MMP-9 and IL-6 increased from baseline in patients receiving anthracyclines at 3- and 6-months post-treatment. Interestingly, these levels correlated with high sensitivity troponin T and I, but not with changes in left ventricular ejection fraction (LVEF), though the study may have been underpowered to detect such a change [36]. In another prospective study of 46 women with breast cancer requiring anthracyclines or trastuzumab, high sensitivity C-reactive protein (hsCRP) increased over a treatment duration of 4 months [37]. In a multicenter prospective study of 78 patients with breast cancer undergoing doxorubicin and trastuzumab therapy, troponin I (TnI), C-Reactive protein (CRP), growth differentiation factor 15 (GDF-15), MPO, placental growth factor (PIGF) and soluble Fms-like tyrosine kinase-1 (sFlt—1) levels increased with anthracycline therapy. However, only elevated levels of TnI and MPO were associated with an increased risk of cardiotoxicity [38]. Conversely, in a prospective study of patients undergoing doxorubicin-containing chemotherapeutic regimens (*n* = 41), noncardiac biomarkers CASP-1 and MPO decreased after administration of chemotherapy, particularly in the high-dose anthracycline subgroup, but were not associated with change in LVEF [39]. A small number of clinical trials have looked at inflammatory markers, particularly IL-6 and ROS, and found that pharmacologic cardioprotection correlated with decreased levels of inflammatory biomarkers compared to placebo groups [40]. It is theorized that, as previously seen in preclinical studies, there is increased inflammation in the setting of malignancy that is reduced with chemotherapeutic treatment. Therefore, these serum markers of inflammation are inextricably linked to both oncologic response and cardiotoxicity. See Table 1 for a detailed summary of studies evaluating the use of inflammatory markers for detection of AIC. Further large-scale studies of multiple inflammatory biomarkers are needed to identify potential clinical biomarkers more specific to AIC.

**Table 1:** Summary of Clinical Studies Investigating Inflammatory Biomarkers in Patients Undergoing Anthracycline-Based Therapy

Study	Study Type	Population	Biomarkers	Change Post-Anthracycline Treatment	Association with Cardiotoxicity
Dessi et al. 2013 [40]	Randomized controlled trial	Cancer patients on epirubicin randomized to treatment with telmisartan (*n* = 25) versus placebo (*n* = 24)	IL-6, TNF-α, serum ROS	↑ — placebo ↔ —telmisartan	Correlation between strain rate on echocardiography and serum IL-6 and ROS observed
Grover et al. 2013 [37]	Prospective cohort trial	Breast cancer patients on anthracyclines and/or trastuzumab (*n* = 46)	hsCRP	↑	No direct cardiotoxicity link reported
Ky et al. 2014 [38]	Prospective cohort trial	Breast cancer patients on anthracyclines and trastuzumab (*n* = 78)	TnI, CRP, GDF-15, MPO, PIGF, sFlt-1	↑	TnI and MPO were associated with increased cardiotoxicity risk
Lakani et al. 2021 [36]	Prospective cohort trial	Breast cancer patients (*n* = 17), matched healthy controls (*n* = 17)	TOP2β, MPO, MMP-2, MMP-9, IL-6,	↑	Biomarkers correlated with high sensitivity troponin T and I; no direct association with LVEF change was observed
Dean et al. 2023 [39]	Prospective cohort trial	Cancer patients (*n* = 41) undergoing anthracycline-based treatment	CASP-1, MPO	↓	Decrease in biomarkers was not associated with change in LVEF

*TOP2β* Topoisomerase II beta, *MPO* Myeloperoxidase, *MMP-2* Matrix Metalloproteinase-2, *MMP-9* Matrix Metalloproteinase-9, *IL-6* Interleukin-6, *hsCRP* High-sensitivity C-reactive protein, *TnI* Troponin I, *GDF-15* Growth Differentiation Factor 15, *PIGF* Placental Growth Factor, *sFlt-1* Soluble Fms-like Tyrosine Kinase-1, *IL-6* Interleukin-6, *TNF-α* Tumor Necrosis Factor-alpha, *ROS* Reactive Oxygen Species, *CASP-1* Caspase-1, *LVEF* Left Ventricular Ejection Fraction

### Targeted Imaging of Inflammation

Traditional imaging of AIC has been primary focused on changes to LVEF. More recently, changes in GLS have been found to be important markers of subclinical cardiac dysfunction and have entered the guideline definition of AIC [3]. However, more specific imaging of inflammation may provide an additional avenue for targeted early detection. In cardiac MRI, an important marker of inflammation can be myocardial edema. Myocardial edema on CMR has been found after anthracycline therapy in about a third of patients at one month and nearly a half of patients at four months. However, this has not been correlated to change in LVEF [37]. Cardiac positron emission tomography (PET) is a promising tool due to the ability to use various radioactive tracers to target specific mechanisms of cardiotoxicity. Indeed, cardiac PET is a well-established tool for diagnosis of inflammatory cardiomyopathies, particularly cardiac sarcoid [41]. In this modality, 18-fluorodeoxyglucose (^18^F-FDG) PET detects regions of increased glucose metabolism which can signal inflammatory activity. In AIC, the impaired mitochondrial phosphorylation and oxidation drives myocytes towards increased glucose metabolism. Increased ^18^F-FDG uptake after anthracycline therapy has been demonstrated in preclinical and retrospective clinical studies. However, prospective studies have not been performed and correlation with cardiotoxicity specifically has not been established. More targeted PET and SPECT tracers are being developed to image specific mechanisms of AIC, including apoptosis (^99m^Technetium-annexin-V) and myocardial necrosis (^111^In-antimyosin)[42].

## Advances in Prevention of AIC: Role of Inflammation

Given the significant morbidity and mortality related to AIC, it is important to identify potential pharmacologic treatments for both cardioprotection and treatment of AIC. Understanding the role of inflammation in this process opens the possibility of novel treatment targets. In some cases, traditional cardioprotective medications have additional pathways that specifically target the mechanisms of AIC, including inflammatory pathways; however, clinical trial data has been mixed.

### Role of Inflammation in Conventional Cardioprotective Therapies for AIC

#### Dexrazoxane

Dexrazoxane, a water-soluble analog of the iron chelating agent ethylenediaminetetraacetic acid (EDTA), was the first FDA-approved treatment for prevention of AIC. Cardioprotective effects of dexrazoxane were initially thought to be via iron chelating effects of the metabolize ADR-925; however, studies of isolated ADR-925 do not reduce cardiotoxicity and inhibition of conversion from dexrazoxane to ADR-925 do not attenuate the cardioprotective effects of dexrazoxane [43–45]. Further studies showed that dexrazoxane depletes TOP2B as a primary mechanism of cardioprotection, which as previously described plays an important role in the inflammatory cascade [44, 46]. Attenuation of TOP2B prevents DNA damage, p53 activation and eventually cell apoptosis. Specific anti-inflammatory pathways have not been identified for cardiomyocytes, however, in a Parkinson’s Disease mouse model, dexrazoxane was found to suppress local and systemic inflammation, as measured by levels of TNF-α and IL-1β [47].

Cardioprotective effects of dexrazoxane during anthracycline therapy have been demonstrated in multiple clinical trials, particularly in children with hematologic malignancies and adults with breast cancer [48–52]. Based on the strength of this data, the FDA approved the use of dexrazoxane specifically in patients with advanced or metastatic breast cancer after a cumulative dose greater than 300 mg/m^2^ of doxorubicin equivalent. Unfortunately, early studies suggested an increased risk of secondary malignancy and concern for reduced antitumor efficacy [53], which has not been reproduced in future studies [54, 55]. Importantly, a large meta-analyses of breast cancer patients undergoing anthracycline-based chemotherapy found that administration of dexrazoxane lead to lower rates of heart failure without a detrimental effect on cancer outcomes [56]. These studies did not specifically evaluate for anti-inflammatory effects.

#### Angiotensin Converting Enzyme Inhibitors (ACEi) and Angiotensin Receptor Blockers (ARBs)

Preclinical studies of angiotensin converting enzyme inhibitors (ACEi) and angiotensin receptor blockers (ARB) have been promising in providing cardioprotection against and treatment of AIC, and in particular its associated inflammation. Doxorubicin increases plasma angiotensin II and local myocardial ACE, which cause direct cardiotoxicity [57]. In rodent models, ACEi and ARBs decreased ROS production, decreased apoptosis, reduced the risk of heart failure, and improved mortality [57, 58]. However, larger clinical trials of ACEi and ARBs have produced mixed results. In a placebo-controlled study of telmisartan, treatment with telmisartan led to decreased levels of IL-6 and ROS, and mitigated changes in myocardial strain at high doses of anthracyclines (> 300 mg/m^2^) [40, 59]. The PRADA (Prevention of Cardiac Dysfunction During Adjuvant Breast Cancer Therapy) trial was a 2 × 2 factorial, randomized placebo-controlled trial of monotherapy and combined candesartan and metoprolol succinate during epirubicin therapy in breast cancer patients. Early results showed candesartan prevented a modest reduction in LVEF compared to other groups [60], but this did not persist at two year follow up [61].

#### Beta Blockers

Beta blockers are a mainstay of treatment for heart failure primarily due to their role in reducing sympathetic activation and neurohormonal upregulation; however, certain beta blockers also have anti-inflammatory and antioxidant effect that increase cardioprotective effects in AIC. In particular, carvedilol and nebivolol have antioxidant properties that reduce ROS [62]. Bisoprolol and carvedilol reduce inflammation and ROS in other etiologies of heart failure with reduced LVEF [63]. A similar anti-inflammatory and antioxidant effect has not been seen with metoprolol. Multiple clinical trials have evaluated cardioprotective effects of various beta blockers, showing a modest benefit to beta blocker therapy, particularly with carvedilol, nebivolol and bisoprolol. In the PRADA study, metoprolol had no efficacy in the prevention of AIC in patients with early breast cancer [61]. In the CECCY (Carvedilol Effect in Preventing Chemotherapy-Induced Cardiotoxicity) trial, the use of carvedilol in women with HER2 negative breast cancer did not lead to a significant difference in the clinical incidence of AIC at six months compared to placebo, but there was a decrease in subclinical markers of cardiac dysfunction including abnormal troponin values and diastolic dysfunction [64]. The OVERCOME trial demonstrated that, in patients with hematologic cancers undergoing high dose anthracycline chemotherapy with possible stem cell transplant, combined enalapril and carvedilol prevented a reduction in LVEF compared to placebo, [65] which was also seen with combined bisoprolol and lisinopril [66]. In a breast cancer population, nebivolol prevented a change in left ventricular end-systolic and end-diastolic diameters, compared to placebo [67]. These results indicate that beta blocker therapy, particularly with carvedilol, nebivolol and bisoprolol, do not prevent clinical incidence of anthracycline-induced cardiotoxicity (change in LVEF > 10% from baseline), but can prevent subclinical changes. Limitations to these studies include small sample sizes, short follow up, low frequency of the outcome, and variations in the patient population and anthracycline exposure.

#### Statins

Statins have pleiotropic effects beyond traditional lipid lowering mechanisms, including anti-inflammatory and antioxidant properties. In a mouse model of AIC, pretreatment with fluvastatin preserved LV function, attenuated oxidative stress, increased expression of antioxidant enzymes, reduced cardiac inflammation via TNF-α expression, and decreased apoptosis compared to controls [68]. Statins also inhibit small Ras homologous (Rho) GTPases, which play a critical downstream role in inflammation as part of the NADPH oxidase complex and stimulation of a pro-inflammatory process [69]. These studies suggest that the cardioprotective effects of statin therapy are mediated by anti-inflammatory, antioxidant, and anti-apoptotic mechanisms [68, 70]. This proposed mechanistic effect was first confirmed with early retrospective studies that suggested that statins prevented reduction in LVEF [71, 72]. More recent randomized, placebo-controlled trials have had mixed results. In a study of high risk patients undergoing anthracycline chemotherapy for hematologic malignancies, high-dose atorvastatin prevented a reduction on LVEF compared to placebo [73]. Notably, an elevation in CRP after initiation of chemotherapy was observed in the control group, but not in the statin group. The STOP-CA (Statins to Prevent the Cardiotoxicity of Anthracyclines) trial was the largest double-blind randomized placebo-controlled trial of atorvastatin for prevention of cardiotoxicity in patients with lymphoma. This study demonstrated a significant reduction of the primary endpoint of reduction of LVEF of > 10% from prior to a final value of < 55% over twelve months [74]. However, the PREVENT (Preventing Anthracycline Cardiotoxicity With Statins) trial of patients with breast cancer (85%) and lymphoma (15%) receiving anthracyclines pretreated with atorvastatin 40 mg daily showed no significant difference in the 24-month change in LVEF [75]. Multiple serum markers of inflammation were reported including CRP, IL-6, and TNF-α, which decreased in both statin and placebo groups at 6- and 24-months post treatment [75]. In the STOP-CA trial, the participants received higher doses of anthracyclines (cumulative median anthracycline dose 300 mg/m^2^ versus 240 mg/m^2^), were older and was limited to patients with lymphoma, representing a higher risk cohort of patients, indicating a likely benefit in the most high-risk patients.

### Targeted Anti-Inflammatory Therapies for Prevention of AIC

Understanding the multiple mechanisms behind AIC are essential to develop new therapies for prevention and treatment of AIC. As seen above, traditional cardioprotective therapies have had mixed or limited responses in clinical trials, so anti-inflammatory therapies may prove to be a promising strategy for prevention of AIC. However, these therapies have only been investigated in preclinical models of disease.

#### Steroids

Only preclinical studies have evaluated the cardioprotective effects of steroid therapy. Dexamethasone reduced the ratio of abnormal cardiomyocytes compared to doxorubicin alone in a mouse model of AIC [76]. Interestingly, the single nucleotide polymorphism rs28714259 has been associated with an increased risk of AIC and, in a human induced pluripotent stem cells (hiPSC)-derived cardiomyocyte cell line, CRISPR-Cas9-mediated deletion of this locus identified glucocorticoid receptor signaling as a key mediator of cardiotoxicity. Pretreatment with dexamethasone in the knock out cell line improved cell viability and contractility, which was not seen in control cells [77].

#### Colchicine

Colchicine is a well-established anti-inflammatory medication that has proven efficacy in inflammatory cardiovascular disease including pericarditis, post-operative atrial fibrillation and atherosclerosis [78–80]. Colchicine irreversibly binds to tubulin to block microtubule polymerization. This disrupts multiple cellular processes, including neutrophil adhesion, TNF-α synthesis and activation of the NLRP3 inflammasome [80]. In preclinical study of in vivo and in vitro doxorubicin induced cardiac dysfunction, low dose colchicine (0.1 mg/kg daily) improved cardiac function compared to placebo [81].

#### NLRP3 Inflammasome Inhibitors

Activation of the NLRP3 inflammasome plays an important role in the inflammatory mechanism of AIC and inhibition of the NLRP3 is an increasingly recognized target for anti-inflammatory therapies. Overexpression of sirtuin 3 (SIRT3) inhibits the NLRP3 inflammasome activation via autophagy, reducing doxorubicin-induced cardiotoxicity [82]. In both a rat model and in vitro cellular model, dihydromyrecetin (DHM), a flavonoid compound, attenuates doxorubicin induced cardiotoxicity by reducing NLRP3 inflammasome-mediated inflammation [83]. Similarly, calycosin, the active component in *Astragalus,* reduces cardiotoxicity through the Sirt1-NLRP3 pathway [84]. Fraxetin, a coumarin from Cortex Fraxini, mitigated both oxidative stress and inflammation through decreased activation of the NLRP3 in a dose-dependent manner. Resveratrol has also been shown to reduce cardiotoxicity via suppression of the NLRP3 inflammasome [27]. Currently, multiple targeted CRID3-based therapies that inhibit the NLRP3 inflammasome are in clinical development in Phase I through Phase III trials in a wide spectrum of inflammatory diseases, including nonalcoholic fatty liver disease, ulcerative colitis, COVID-19-associated pneumonia, and neurodegenerative diseases. None have yet been trialed in prevention of AIC but may be promising therapies in the future.

#### Cytokine Receptor Blockers

In addition to NLRP3 inflammasome targeted therapies, there are more downstream targets anti-inflammatory therapies that target cytokines, like anakinra (IL-1R antagonist), canakinumab (IL-1β neutralizing antibody) and rilonacept (soluble receptor that binds IL-1β and IL-1α). None have been studied in this population but are currently being evaluated in the cardiovascular sphere, notably in coronary artery disease [85] and heart failure [86].

## Application to Other Forms of Cancer Therapy Related Cardiac Dysfunction

The scope of this review has focused on the mechanism of AIC because it is the most well recognized and well-studied form of CTRCD and the mechanisms of AIC can serve as a model to understand other forms of CTRCD. For example, pyroptosis, a key driver of the cycle of inflammation as a result of cell death, has been identified in other anticancer agents beyond anthracyclines because it is often a key driver of the anticancer properties; however, the off-target effects in the myocardium contribute to cardiotoxicity [30]. Similarly, other chemotherapy agents have been shown to activate the NLRP3 inflammasome in drug-induced toxicity of other organs. For example, cisplatin increases Nox4 with downstream activation of NLRP3 inflammasome and leads to drug-induced nephrotoxicity [87]. Bleomycin induced pulmonary toxicity occurs via HIF-1α induced NLRP3 inflammasome activation. Additionally, both bortezomib- and paclitaxel-induced neurotoxicity are partially mediated by activation of NLRP3 inflammasome [87]. By understanding mechanisms of toxicity that are common between multiple chemotherapeutic agents, hopefully more universal cardioprotective strategies can be devised.

In addition to cardiotoxicity from chemotherapy, the rise of immunotherapy has highlighted the important role of myocardial inflammation in the development of CTRCD. While this review has focused on the role of the innate immune system, immune checkpoint inhibitors primarily cause cardiotoxicity via alterations in the adaptive immune system, particularly T-cells. Importantly, the rare, but potentially fatal condition immune checkpoint inhibitor myocarditis occurs as a result of the loss of normal checks on inflammation leading to a robust inflammatory infiltration within the myocardium and resulting cardiac dysfunction [88].

The above discussions of the role of cytokines in both triggering an intracellular inflammatory cascade and alterations in cardiac contractility have important implications in cardiotoxicity from new T-cell mediated immunotherapies, including CAR T-cell therapy. With CAR T-cell therapy, there is a well-documented side effect of cytokine release syndrome that is associated with cardiotoxicity [89]. Treatment with IL-6 targeted therapies can mitigate this cardiotoxicity.

## Conclusions

CTRCD occurs because of multiple complex and overlapping mechanisms that lead to cardiotoxicity. In the case of AIC, inflammation plays an important role and is part of a cycle whereby initial cardiac damage triggers an inflammatory response which in turn causes further cardiotoxicity. AIC-induced inflammation provides an important target for diagnosis and treatment of AIC, though the exact role deserves further study. An improved understanding of this mechanism may lead to novel methods of toxicity detection and prevention to improve the care of all patients with cancer. Additional research and expanded clinical studies are needed to further elucidate the role of inflammation in chemotherapy induced cardiotoxicity.

## Key References

- Krysko DV, K.A., Krysko O, Heyndrickx L, Woznicki J, Bogaert P, Cauwels A, Takahashi N, Magez S, Bachert C, and Vandenabeele P., *TLR-2 and TLR-9 are sensors of apoptosis in a mouse model of doxorubicin-induced acute inflammation.* Cell Death Differ, 2011. **18**: p. 1316–1325
  - **Findings demonstrate that doxorubicin induces an immunogenic form of apoptosis that upregulates the innate immune system via toll-like receptors (TLRs)**
- Lakhani, H.V., et al., *Detecting early onset of anthracyclines-induced cardiotoxicity using a novel panel of biomarkers in West-Virginian population with breast cancer.* Scientific Reports, 2021. **11**(1).
  - **Findings demonstrate that a novel panel of biomarkers, including inflammatory biomarkers, correlated with myocardial damage from AIC, as measured by troponin T and troponin I.**
- Jong, J., J.R. Pinney, and R.R.S. Packard, *Anthracycline-induced cardiotoxicity: From pathobiology to identification of molecular targets for nuclear imaging.* Frontiers in Cardiovascular Medicine, 2022. **9**.
  - **This review provides an overview of nuclear imaging techniques for detecting anthracycline-induced cardiomyopathy (AIC) and evaluates the evidence supporting the use of novel tracers for this purpose, including those capable of identifying inflammation associated with AIC.**
- Sun, Z., et al., *Dihydromyricetin alleviates doxorubicin-induced cardiotoxicity by inhibiting NLRP3 inflammasome through activation of SIRT1.* Biochemical Pharmacology, 2020. **175**: p. 113,888
  - **Findings demonstrate that dihydromyricetin can alleviate AIC via inhibiation of the NOD-like receptor protein 3 (NLRP3) inflammasome.**

## Data Availability

No datasets were generated or analysed during the current study.
